# The impact of the Covid-19 pandemic restrictions on children entering and leaving care in Scotland

**DOI:** 10.23889/ijpds.v8i2.2207

**Published:** 2023-09-14

**Authors:** Joanna Soraghan, Gillian Raab, Patricio Troncoso

**Affiliations:** 1 CELCIS, University of Strathclyde, Glasgow, United Kingdom; 2 University of Edinburgh, Edinburgh, United Kingdom; 3 Heriot-Watt University, Edinburgh, United Kingdom

## Objectives

The Covid-19 pandemic caused huge upheaval across the world, and the Scottish children’s social care sector was not exempt from this. This study explored the extent to which the Covid-19 pandemic restrictions impacted on the rate at which children and young people were entering and leaving care in Scotland.

## Method

Analysis was conducted on the Scottish Government’s ‘Longitudinal Looked After Children’ dataset which contains information on the care journeys of all children who were ‘looked after’ in Scotland between April 2008 and July 2021. Through a descriptive analysis, the study determined how the pandemic restrictions impacted the rates of children entering care, the rates of children leaving care, and the stability and duration of care placements at this time.

## Results

During the initial year of the pandemic, there was a marked reduction in the number of children both entering (38%) and leaving (22%) care when compared to the year prior. The reduction in entries to care were less notable for infants under the age of 1 than it was for older age groups. Fewer children entered care under compulsory measures, with an increased proportion entering through ‘voluntary’ Section 25 measures. The impact on entries to care and exits from care varied substantially across local authorities in Scotland. The stability and duration of children’s placements were also impacted, with children moving placements less and staying in care for longer periods of time.

## Conclusion

The pandemic restrictions had a substantial impact on the interactions of children and young people with the ‘care system’. At a time where there is great focus on improving the experience of young people in care within Scotland, it is important that lessons are learned and implemented for future disruptive events.

